# A hybrid artificial intelligence approach for modeling the carbonation depth of sustainable concrete containing fly ash

**DOI:** 10.1038/s41598-024-62737-1

**Published:** 2024-05-25

**Authors:** Ramin Kazemi

**Affiliations:** Department of Civil Engineering, Hakim Sabzevari University, Sabzevar, Iran

**Keywords:** Civil engineering, Cementitious composites, Carbonation depth, Artificial intelligence, Sustainable concrete, Fly ash, Civil engineering, Materials science, Mathematics and computing

## Abstract

One of the major challenges in the civil engineering sector is the durability of reinforced concrete structures against carbonation during the physico-chemical process of interaction of hydrated cementitious composites with carbon dioxide. This aggressive process causes carbon penetration into the reinforcement part, which affects the behavior of the structure during its lifetime due to corrosion risk. A countermeasure is using alternative cementitious materials to improve concrete texture and resist increased carbonation depth (CD). Considering that the CD test requires a long time and a skilled technician, this study strives to provide an alternative approach by moving from traditional laboratory-based methods towards artificial intelligence (AI) techniques for modeling the CD of sustainable concrete containing fly ash (CCFA). Despite the development of single AI models so far, it is undeniable that utilizing metaheuristic optimization techniques in the form of hybrid models can improve their performance. To this end, a new hybrid model from the integration of biogeography-based optimization (BBO) technique with artificial neural network (ANN) is developed for the first time to estimate the CD of CCFA. The error distribution results revealed that 59% of the ANN predictions had errors within the range of (− 1 mm, 1 mm], while the corresponding percentage for the ANN-BBO predictions was 70%, indicating an 11% reduction in the prediction errors by the proposed hybrid model. Furthermore, A10-index highlighted a performance improvement of 78% for the hybrid model, which met the closeness of the predicted values to the observed ones, so that the value of this index for models of ANN and ANN-BBO was 0.5019 and 0.8947, respectively. Analyzing the cross-validation confirmed the reliability and generalizability of the developed model. Also, the three most influential variables in estimating the CD were exposure time (27%), carbon dioxide concentration (22%), and water/binder (18%), respectively. Finally, the superiority of the ANN-BBO model was verified by comparing it with previous studies’ models.

## Introduction

Exposure to aggressive environments can widely affect the safety and durability of reinforced concrete structures (RCSs). One of the main deterioration mechanisms affecting the durability of RCSs is the carbonation phenomenon^1^. This phenomenon is caused by the physico-chemical process of concrete affected by prolonged exposure to carbon dioxide (CO_2_) permeation from the ambient air through concrete micro-defects with hydrated cement composites, which leads to the production of calcium carbonate (CaCO_3_) and water (H_2_O). During this process, the release of hydroxides (OH^−^) from calcium hydroxide (Ca(OH)_2_) and the formation of free calcium (Ca^2+^) leads to a gradual decrease in the potential of hydrogen (pH) range of the pore solution below 9 in concrete^2,3^.

**Figure 1:**
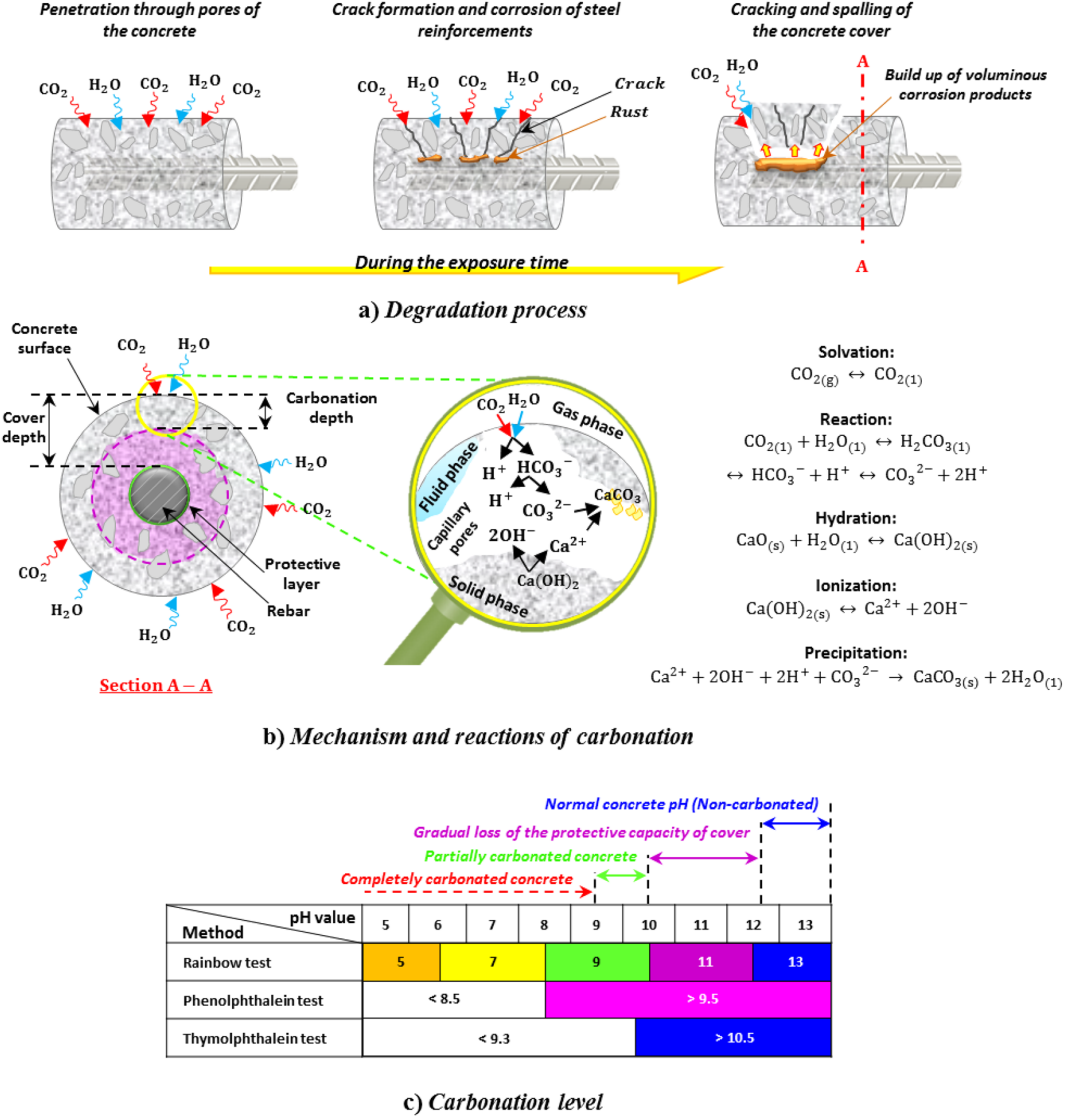
An in-depth overview of the concept of concrete carbonation. (**a**) Degradation: it is a process over time that starts with the penetration of CO_2_ and H_2_O through the concrete pores and continues with the formation of cracks and corrosion of steel reinforcements, causing cracking and spalling of the concrete cover; (**b**) Mechanism and reactions: it shows in detail the chemical reactions that occur in the three phases of fluid (H_2_O), solid (Ca(OH)_2_) and gas (CO_2_) that lead to the formation of CaCO_3_; and (**c**) Diagnosis^3^: it specifies the carbonation level for different methods regarding pH value (this figure was created by the author using Microsoft PowerPoint 2016).

Its effect in unreinforced concrete appears as the decrease in capillary absorption due to the blocking of carbonate pores (the increase in impermeability) and, thereby, the increase in the mechanical property^4^. Nevertheless, the carbonation effect is considered a severe threat to RCSs because reducing alkalinity endangers the protective role of concrete and the protective layer on rebar, resulting in corrosion risk^5^. As the carbonated zone reaches the rebar and depassivate it, electrochemical corrosion in the vicinity of oxygen and moisture starts to produce rust and the following damages to the concrete through spalling and cracking. As a result, with its progress, the mechanical properties of the structure diminish^6^. At a glance, Fig. 1 provides an in-depth overview of the concept of concrete carbonation.

In general, the influencing factors on the carbonation process of RCSs can be classified into two main categories, external and internal.

The crucial external factors include CO_2_ concentration, exposure time, and relative humidity, also known as environmental factors. It is not far-fetched that increasing CO_2_ concentration and exposure time result in a more aggressive environment and thereby endanger the long-term durability of RCSs^7,8^. The environment's relative humidity (RH) significantly affects the speed of the carbonation reaction, and its peak effect is achieved at an RH of 50–70% for normal concrete. The carbonation process slows down for dry concrete due to the lack of sufficient moisture and for saturated concrete due to water's blocking effect and the subsequent possible reduction of CO_2_ entry^9^. In terms of internal factors, constituent materials and mix design influence the carbonation process, and their effect appears in the formed texture of cementitious materials^2^. The porous texture reduces the resistance to penetration-in other words, it facilitates the entry of CO_2_ into itself, resulting in a higher carbonation level.

**Figure 2:**
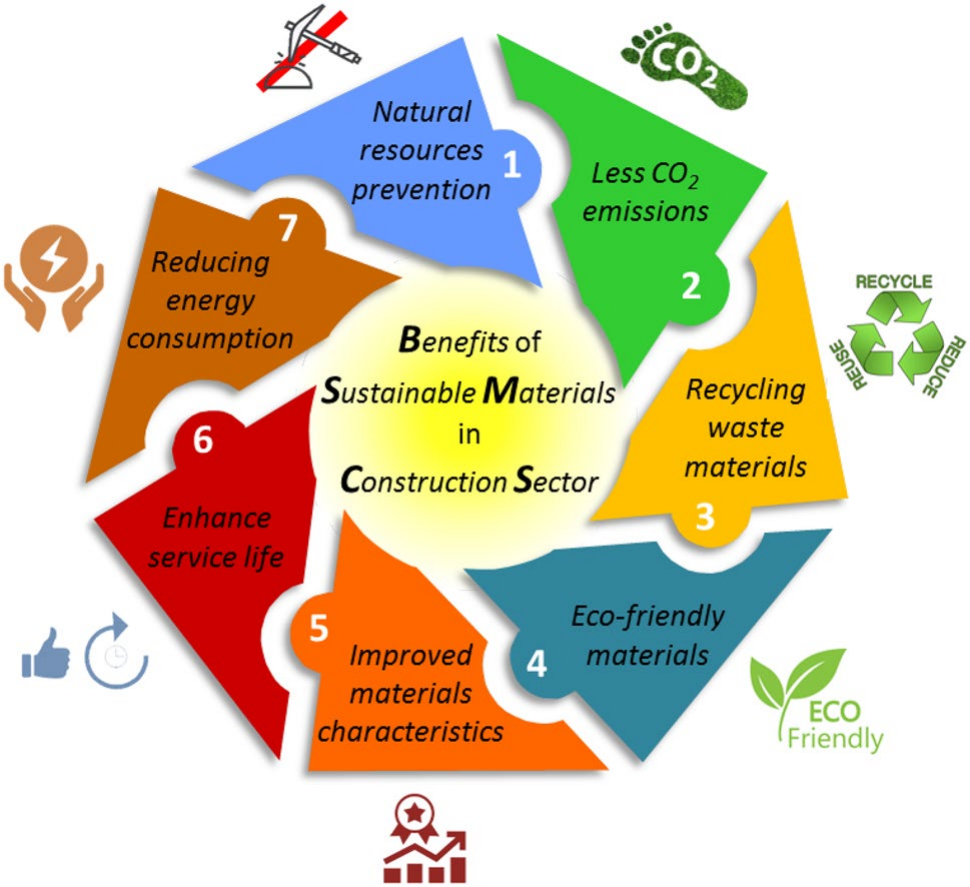
The role of alternative cementitious materials in the sustainable construction sector. Each of the numbers 1–7 in the figure depicts a major advantage of utilizing alternative cementitious materials for sustainable development (this figure was created by the author using Microsoft PowerPoint 2016).

Since controlling external factors seems complicated, a countermeasure is to use by-products/waste materials as alternative cementitious materials. Using these materials reduces carbon emissions by reducing cement consumption and increases the durability and properties of concrete by improving its texture. The benefits of utilizing alternative cementitious materials in the sustainable construction sector are depicted in Fig. 2.

As an alternative supplementary cementitious material, fly ash (FA) is a by-product of coal combustion that adds sustainability to concrete by significantly mitigating carbon emissions^10^. The lower permeability characteristic of concretes containing FA (CCFA) leads to improved durability and resistance to degradation caused by exposure to corrosive environments^11^. Over recent years, numerous experimental studies have been carried out to investigate the carbonation depth (CD) of CCFA. By scrutinizing the literature, it can be found that the effect of FA on concrete carbonation is still an open research topic with the potential for further research, as existing studies have presented different carbonation effects with various FA percentages. Atis^12^ reported that concrete with 70% FA replacement accelerates the carbonation process, while 50% replacement resulted in less or similar carbonation to the reference concrete. In other studies, replacing FA with 10%^13^ and 30%^14^ was reported for the same performance as normal concrete. In the last years, several studies^15–17^ have presented mathematical models to estimate the CD. It is worth noting that the nature of these studies is based on mathematical relationships defined between variables to achieve the desired result in the form of an equation, which limits our ability to generalize the models. In other words, changes in mixture or environmental condition variables require modification of these models. Besides, the investigation of previous studies^16,17^ reveals that their models were often developed to handle a limited number of data records, reducing their reliability. It is undeniable that adopting the traditional laboratory-based methods and mathematical models to determine the CD faces challenges such as (i) the time-consuming and high costs of providing test conditions, (ii) the necessity for re-testing by changing external (e.g., CO_2_ concentration or relative humidity) or internal (e.g., raw materials or mix design) factors, and (iii) inability to fully clarify the relationship between factors and CD arising from the limited number of prepared mixtures and tested samples. In that vein, an alternative and effective strategy is to apply an artificial intelligence (AI) approach aiming to overcome the mentioned challenges and reduce the dependence on laboratory methods as well as achieve reliable results.

In recent years, utilizing AI techniques has been considered a promising approach for modeling concrete properties^18–23^. AI techniques can figure out complex relationships between multiple variables in advanced concrete technology problems without explicitly knowing its underlying processes^24–27^. Among the well-known AI-based forecasting techniques, the artificial neural network (ANN) model is identified as a powerful tool for its ability to (i) identify patterns, (ii) establish an effective relationship between variables, (iii) model non-linear statistical data, (iv) tolerate errors, and (v) flex to solve complex real-world issues^28^. These features have made the ANN model for predicting the properties of sustainable cementitious materials gain wide acceptance^29,30^. With the progress of AI techniques, metaheuristic optimization techniques have been developed as hybrid models to strengthen single prediction models and minimize weaknesses by figuring out efficient solutions and boosting accuracy^31–34^. Indeed, applying the hybrid AI approach can provide a more accurate model with more reliable results. Recently, a few AI-based studies have been developed for estimating the CD of CCFA^35–38^.

## Research gaps and objectives

Scrutinizing the literature background revealed that there are still gaps that need to be overcome to achieve more accurate and reliable models. Hence, the specific gaps in the literature that this study seeks to address include the following:

- Despite the superiority of hybrid AI models, most of the models proposed by the literature so far were single models. However, hybrid models can overcome single models' overfitting and local minimum problems by searching for a wider solution space.
- In none of the existing models, data were divided based on a three-way holdout method for hyperparameter tuning. If the goal is to achieve a model that can be generalized to future data, the two-way holdout method utilized in the literature is inefficient.
- Without a doubt, it is essential to adopt a comparative analysis with previous models to verify the developed model and confirm its superiority and uniqueness, an issue that most of the existing research lacks.

Accordingly, it calls for the pursuit of filling these gaps. To this end, this study deals with one of the most important issues in civil engineering, i.e., the carbonation of concrete, and its significance in that its effect on reinforced concrete structures is considered one of the main destruction factors. In view of this, using alternative cementitious materials in the form of sustainable concrete can be an effective solution to reduce carbonation depth and mitigate negative environmental effects due to less cement consumption. Since adopting the traditional laboratory-based methods for CD determination is time-consuming and requires re-testing with changing materials and conditions, it is time to develop AI-based techniques to provide an alternative approach and minimize the reliance on laboratory activities. In this regard, this study attempts to bridge the above research gaps in AI-based studies to estimate the CD of CCFA with the following main objectives:

- Utilizing the metaheuristic algorithm of biogeography-based optimization (BBO) combined with ANN to develop a new hybrid model for estimating the CD of CCFA.
- Applying the three-way holdout method to divide the data to achieve a generalizable model for future research.
- Establishing a comparative analysis between the proposed model's performance in the current study and previous studies to verify its superiority over the literature models in a more accurate estimation of the CD of CCFA.

**Figure 3:**
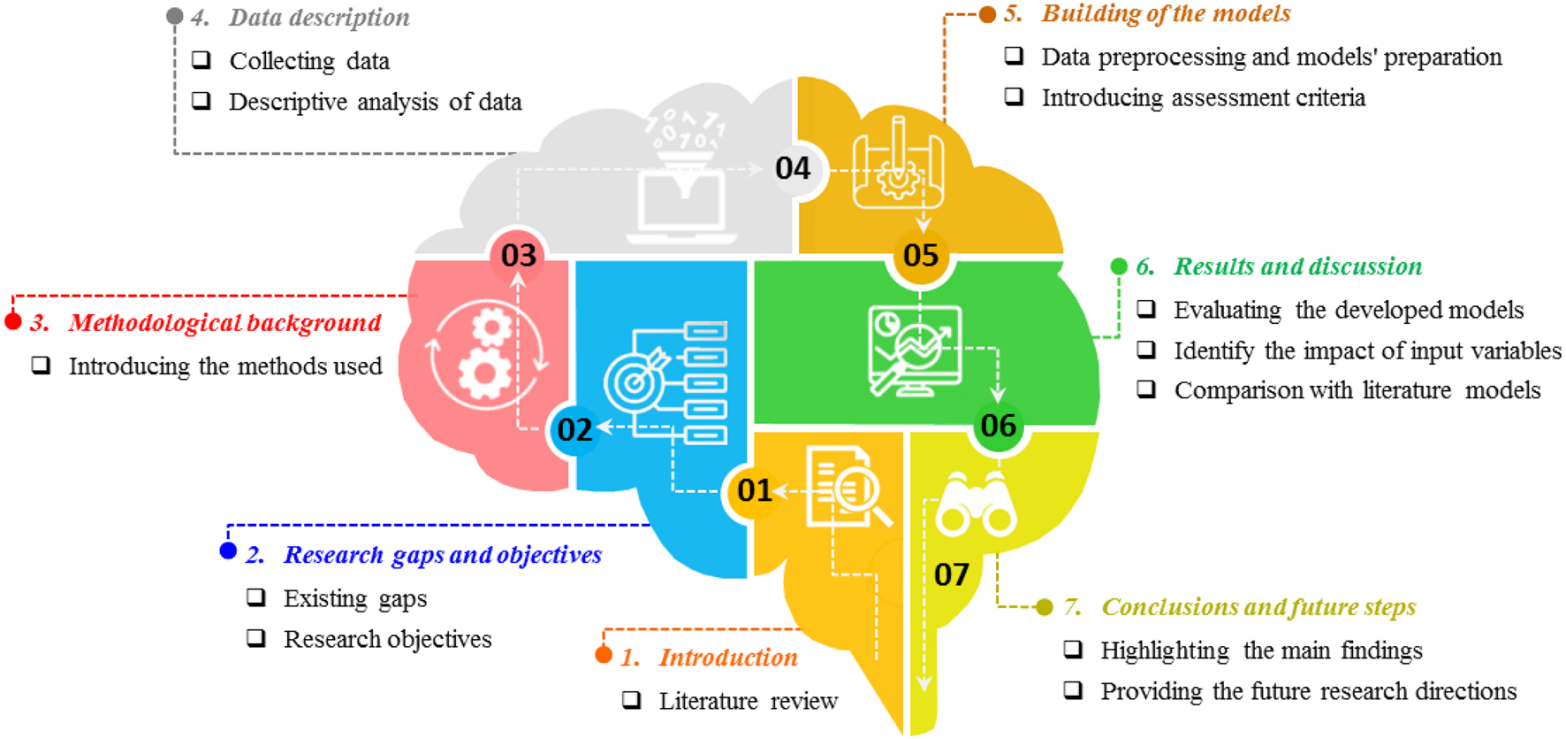
The schematization of the research roadmap in this study includes 7 sections: (1) literature review, (2) determination of research gaps and objectives, (3) introduction of used methods, (4) data description, (5) model construction, (6) presentation of results and discussion, and 7) conclusions and future steps (this figure was created by the author using Microsoft PowerPoint 2016).

At a glance, the schematization of the research roadmap in this work is shown in Fig. 3.

## Methodological background

### Artificial neural network (ANN)

**Figure 4:**
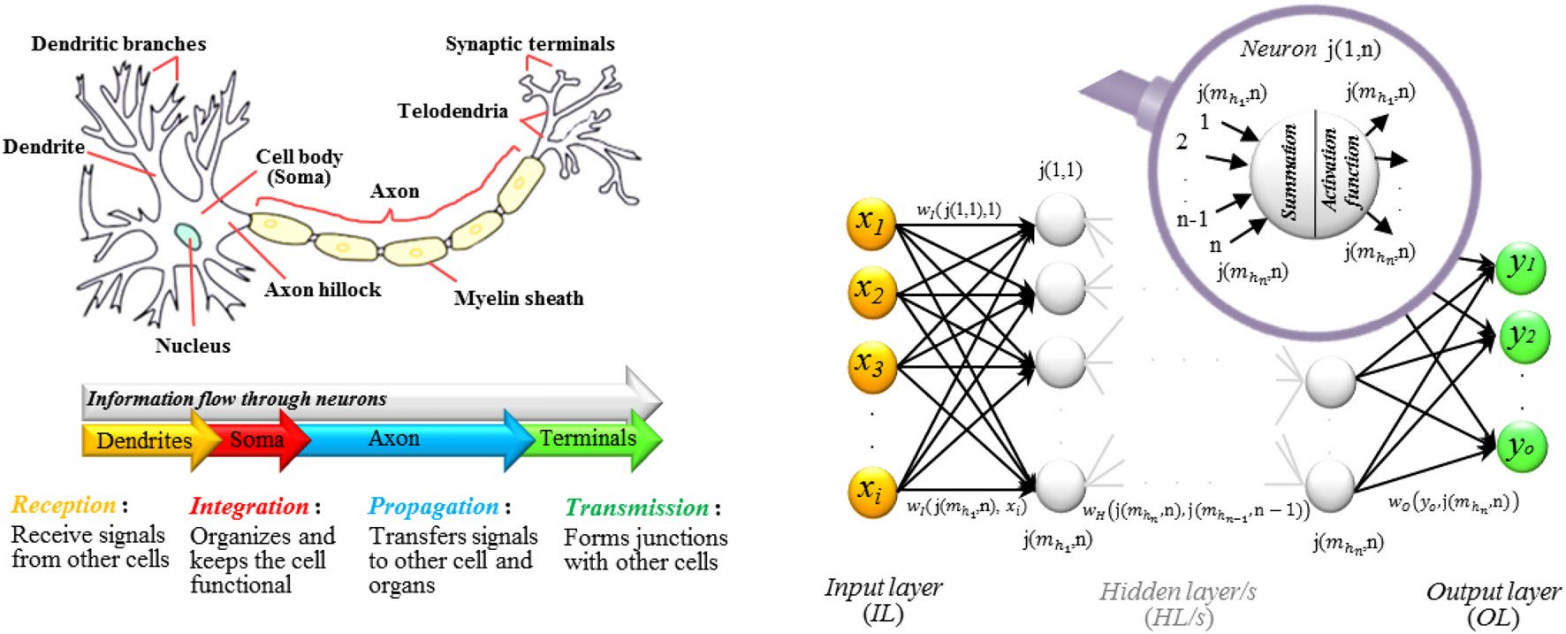
The structure of the ANN model is based on biological neural networks in the human brain and how information is sent and communicated between brain neurons to make a decision (this figure was created by the author using Microsoft PowerPoint 2016).

The idea behind developing the ANN model was taken from the biological neural networks in the human brain^39^. The ANN framework comprises interconnected neurons arranged into input, hidden, and output layers. Notably, there is a structured hierarchy without direct links between neurons in the same layer. So that the input layer corresponds to the quantity of the input variables. Meanwhile, the output layer of the ANN indicates the outcomes as a reflection of the modeling goal. The middle layers in the ANN structure are known as hidden layers that are in charge of receiving, processing, and transmitting information, which is a way of mimicking the neural processes in the biological system of the human brain. Indeed, information flows through neurons by receiving signals (inputs) through the dendrites. After that, the analysis of received signals, i.e., the metabolic activities of the cell, is done in the cell body (weight assignment through activation function). The signals are then transmitted along the axon and finally to the next neuron via axon terminals^40^. Figure 4 shows the inspired structure for the ANN model. The output variable of ANN model is calculated as follows:

1 $$Output_{j} = f\left( {\mathop \sum \limits_{i = 1}^{n} Input_{i} \times weight_{ij} + bias_{j} } \right)$$

where: *f* = activation function, *n* = no. of input variables.

### Biogeography-based optimization (BBO)

**Figure 5:**
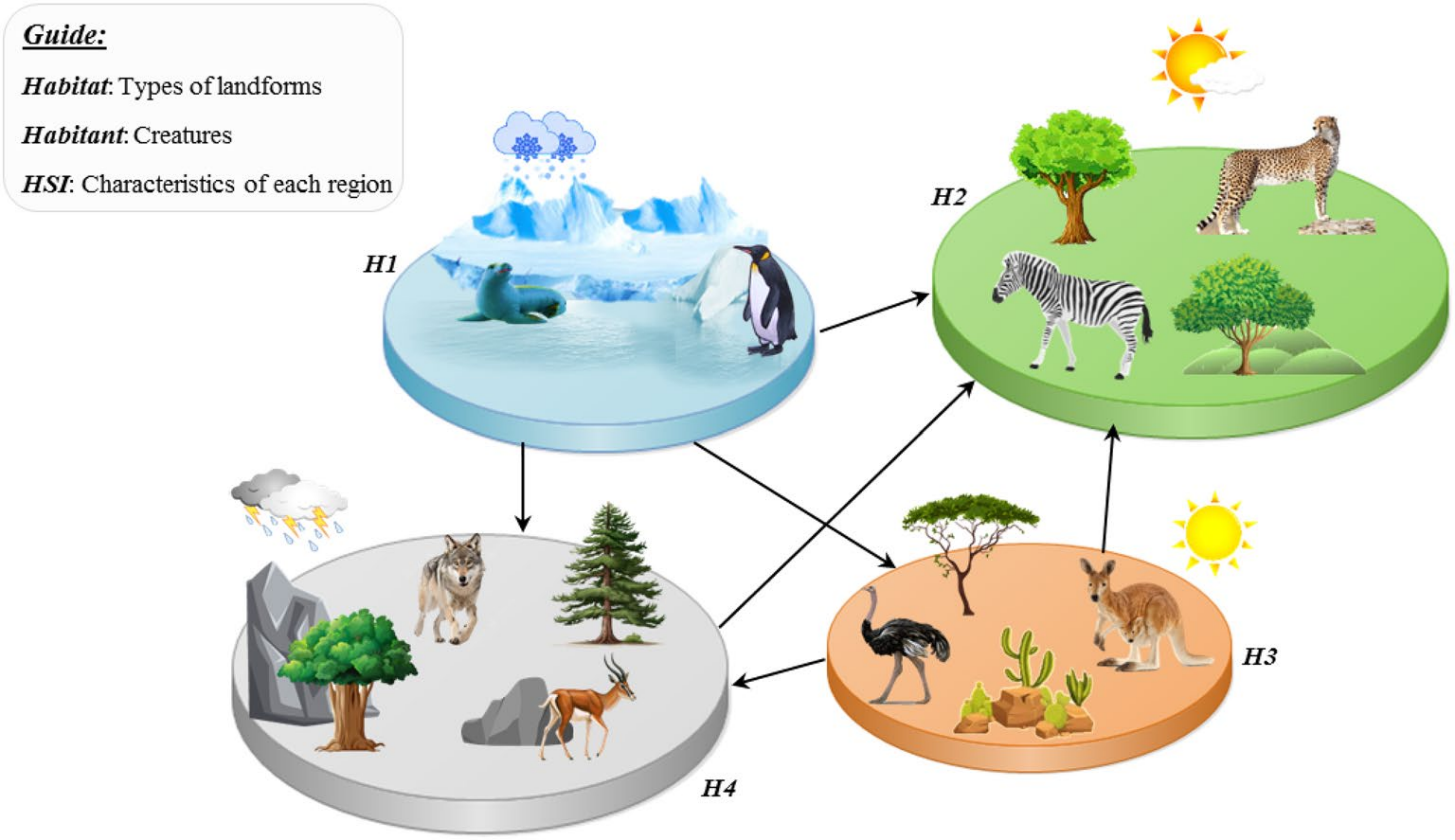
The structure of the BBO method is based on the distribution of species in geographical landscapes and changes in the population of habitats according to the habitat suitability index (this figure was created by the author using Microsoft PowerPoint 2016).

The BBO method is developed as an optimization algorithm inspired by the distribution of species in geographic landscapes. This heuristic optimization technique is based on the processes of immigration, emigration, and mutation by different species (habitants) in biological ecosystems (habitats) to figure out optimal solutions for complex problems^41^. Its ultimate goal is to achieve a stable situation (i.e., the evolution of ecosystems) concerning different species in habitats (e.g., predator and prey) and the effects of their migration and mutation^42^. Potential solutions are proposed under the idea of migration to habitats. So that these solutions increase the quality of the population in successive iterations through the continuous exchange of information between different habitats. The probability of migration to a new habitat is affected by the habitat suitability index (HSI), while the more suitable the habitat is, the higher the HSI. The mutation process allows for a wider solution space, thus enabling better potential solutions^43^. Hence, nature takes steps with these concepts towards improving the balance between different ecosystems, leading to the evolution of initial random solutions. Figure 5 illustrates the inspired structure for the BBO method.

The basic functions of the BBO method are defined as follows:

2 $${\text{Immigration}}_{k} = {\text{Immigration}}_{max} \left( {1 - \frac{k}{{k_{max} }}} \right)$$

3 $${\text{Emigration}}_{k} = {\text{Emigration}}_{max} \frac{k}{{k_{max} }}$$

4 $${\text{Mutation}}_{k} = {\text{Mutation}}_{max} \left( {1 - \frac{{P_{k} }}{{P_{max} }}} \right)$$

where: *k* and *k*_*max*_ = no. of current habitants and No._max_ possible of habitants that the habitat can support, *P*_*k*_ and *P*_*max*_ = mutation probability of the *k*th habitat and argmax(*P*_*k*_).

After familiarizing oneself with the optimization technique of the BBO, one can examine its advantages compared to the existing techniques, which is not without merit. The superiority of BBO is due to the nature of this technique, which has migration and crossover strategies to avoid local minima^42^. These operators cause abrupt changes in the candidate solutions that significantly enhance the exploration ability of BBO. Also, Mirjalili et al.^42^ investigated the performance of the BBO with five other techniques-particle swarm optimization, genetic algorithm, ant colony optimization, evolutionary strategies, and probability-based incremental learning and the results showed that these five techniques do not have operators that promote sudden changes in the candidate solutions, so it is trapped in local minima more often than BBO. It indicates the superiorities of simplicity, flexibility, and computational efficiency, as well as its stochastic nature, which does not need objective function derivatives. It has been proven that BBO is a competitive algorithm in the field of optimization that solves a wide variety of real-world issues^44,45^.

## Data description

**Figure 6:**
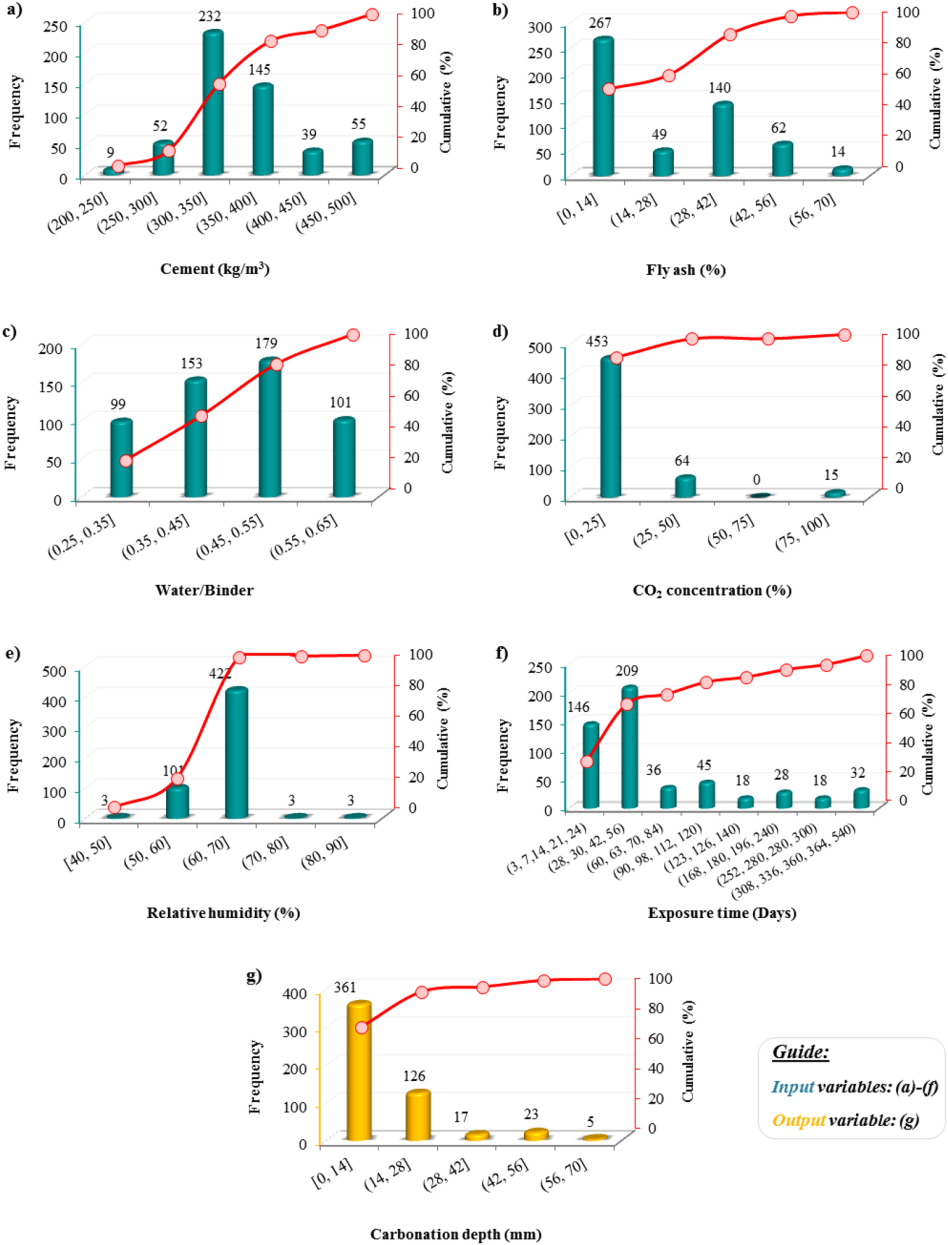
Frequency histogram of input variables (**a**–**f**) and output variable (**g**). Bar charts represent frequency values and line graphs represent cumulative values.

This study collected a comprehensive dataset of 532 data records from 11 literature sources to model the CCFA CD^7,13,36,46–53^ (see Supplementary File). In the performed models, six input variables, consisting of experimental parameters such as cement (as per kg/m^3^), fly ash (as per %), water/binder, and environmental condition parameters such as CO_2_ concentration (as per %), relative humidity (as per %), exposure time (as per day) were considered for the prediction of the carbonation depth (as per mm). Frequency histograms of variables are shown in Fig. 6. As depicted in this figure, about 80% of the mixtures in the database contain cement and fly ash in the range of (200, 400] kg/m^3^ and [0, 42] percentage, respectively. Regarding the water/binder, over 80% of the data records fall within the range of (0.25, 0.55]. Regarding CO_2_ concentration and relative humidity, about 80% of data records fall between 0–25% and 40–70%, respectively. Also, the most frequent exposure time for testing the specimens is 28–56 days. Meanwhile, the database shows the highest recorded CD frequency in the 0–14 mm range. The descriptive statistics of the variables are presented in Table 1.

**Table 1:** Descriptive statistics of the variables.

Descriptive statistics	Variables						
	Cement	Fly ash	Water/binder	CO_2_ concentration	Relative humidity	Exposure time	Carbonation depth
Type of variable	Input	Input	Input	Input	Input	Input	Output
Unit	kg/m^3^	%	–	%	%	Days	mm
Range	222–500	0–70	0.28–0.65	0–100	40–90	3–540	0–61.5
Average	365.92	18.61	0.47	15.07	64.98	76.47	12.33
Standard deviation	61.44	20.22	0.09	20.66	5.13	98.15	12.44
Kurtosis	− 0.26	− 0.83	− 0.95	5.54	5.46	4.74	3.14
Skewness	0.42	0.62	0.09	2.32	− 0.37	2.20	1.73

**Figure 7:**
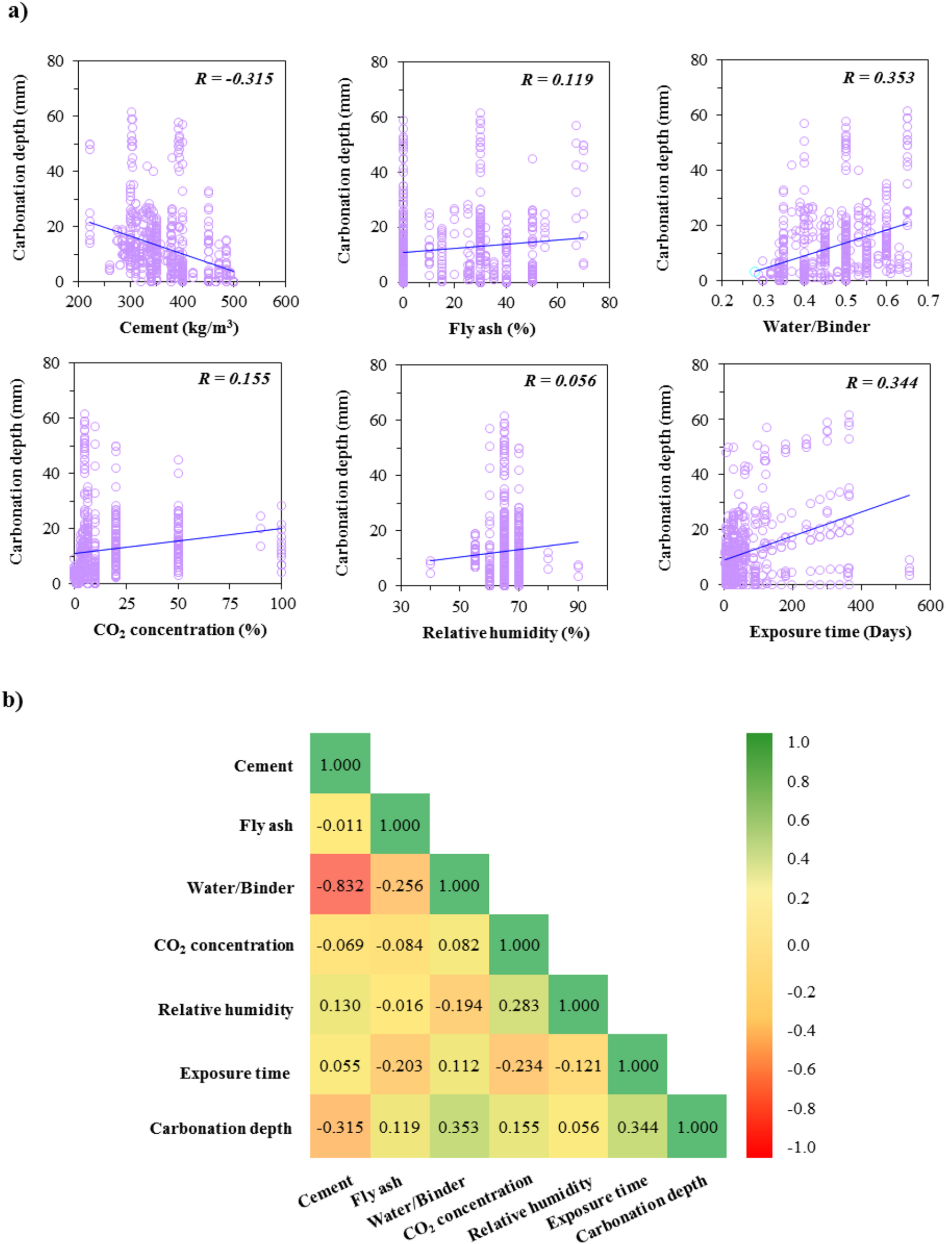
(**a**) Scatter plots of input variables vs. output variable and (**b**) Pearson’s correlation coefficient.

Predictive techniques' accuracy highly relies on choosing the input as an independent variable to estimate the output. AI techniques try to figure out a relation between these variables, so selecting suitable and effective variables can achieve more accurate models. In this vein, a sensitivity analysis of the linear correlation between inputs and output (i.e., CD) has been conducted. As depicted in Fig. 7a, two variables of water/binder and exposure time have the strongest correlation with CD, followed by cement.

Figure 7b illustrates Pearson’s correlation coefficient between variables, consistent with linear correlation. The water/binder and exposure time depict the most correlation with CD. On the other hand, Golafshani et al.^54^ recommended that if the correlation coefficient of any pair of input variables is less than 0.8, it can reduce the risk of multicollinearity in this regression problem. According to Fig. 7b, all correlation coefficients between input variables except water/binder with cement met less than this criterion, which showed no potential bias to the model. Regarding the correlation of water/binder with cement, it should be noted that its negative sign means that when one changes, the other changes in the opposite direction. Also, the reason for the slight difference in its value with the mentioned criterion is that cement exists indirectly in the water/binder. On the other hand, the water/binder variable plays an important role in concrete issues, which is considered an essential modeling variable. Nevertheless, Pearson’s correlation indicates a poor linear relation between variables since there is a nonlinear and complex relation between variables. This reveals the need for more complicated tools like AI to find this relationship and estimate the CD with appropriate accuracy.

## Building of the models

### Data preprocessing and models' preparation

**Figure 8:**
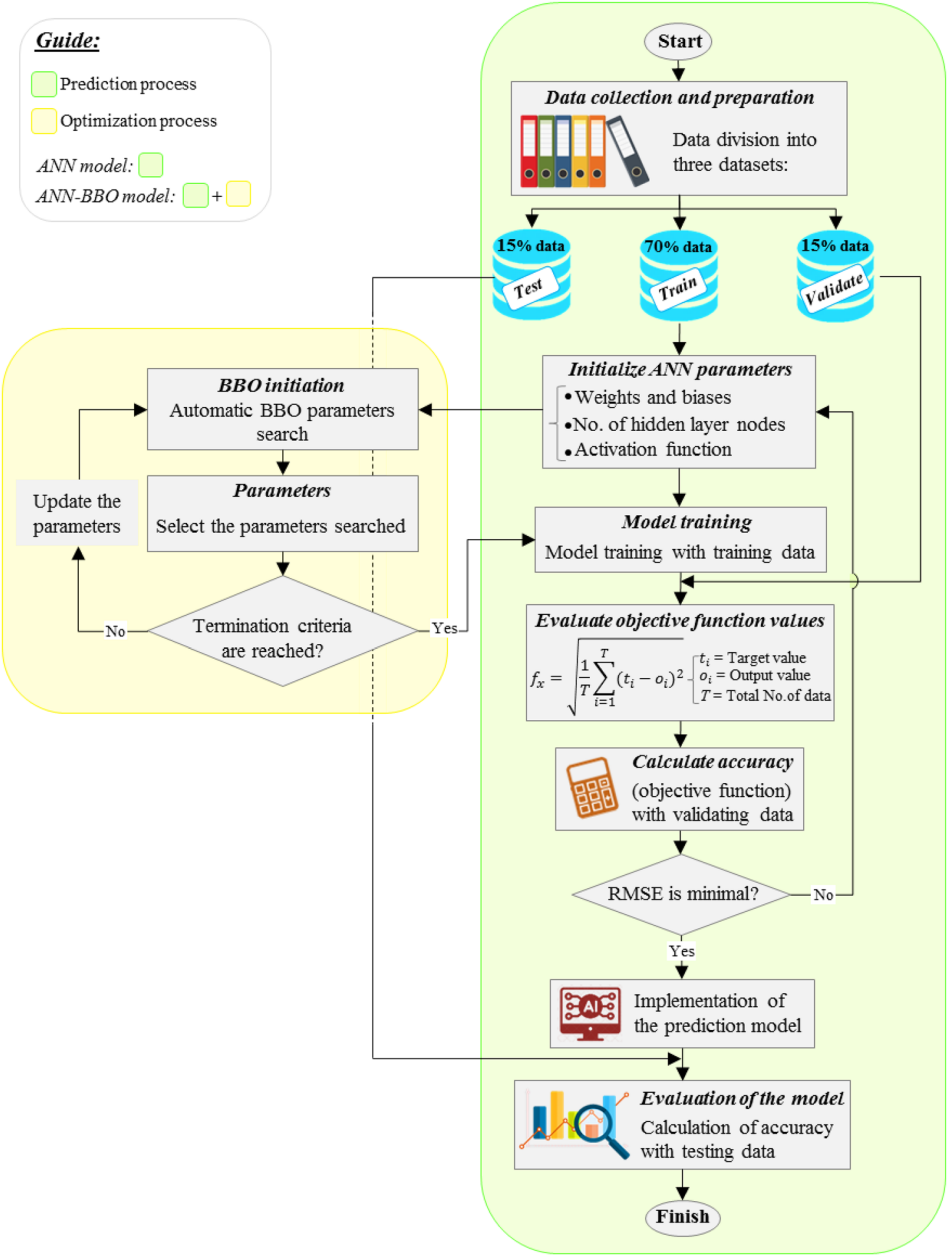
The flowchart of the implementation process of the models of ANN and hybrid ANN-BBO (this figure was created by the author using Microsoft PowerPoint 2016).

The current study seeks to fulfill the application of AI techniques for modeling the CD of CCFA. For this purpose, two AI models of ANN and hybrid ANN-BBO have been developed. It can provide useful insight into the performance of the hybrid ANN model with the metaheuristic optimization technique-BBO compared to its single model of the ANN. This metaheuristic technique helps to determine the optimal parameters, leading the prediction model to improve more accurate results with minimum error. The implementation process of two models of ANN and hybrid ANN-BBO is detailed in the form of a flowchart in Fig. 8. The main difference between the implementation of these two models is the integration of an optimization section by utilizing the BBO technique for the hybrid model, which leads to optimizing the prediction parameters during the implementation process by updating. This feature is considered a significant advantage of the hybrid model compared to the individual model. In this regard, the evaluation results of the selected hyperparameter tuning are presented in the subsection “Determination of the final structure of models” (see Figs. 10, 11 and Table 3).

The first step in data preprocessing is dividing them (532 data points) in the form of three datasets-training dataset (*TrDs*), validation dataset (*VaDs*), and testing dataset (*TeDs*) with proportions of 70% (372), 15% (80), and 15% (80), respectively. This segmentation is aimed at training the model (on *TrDs*), preventing overfitting (on *VaDs*), and testing the model after execution (on *TeDs*). The next step is the normalization of the data. After data division, the next indispensable step is normalizing the data. This step remarkably affects the development of the model, as it converts variables with different boundaries into unitless variables in a certain boundary and decreases the effect of the difference of higher values on lower ones^55^. To this end, the variables were normalized into the range of (− 1, 1) to make them in accordance with the boundaries of the hyperbolic tangent transfer function used through the equation below:

5 $$V_{ normalized} = \frac{{2\left( {V_{i} - V_{minmum} } \right)}}{{V_{maximum} - V_{minmum} }} - 1$$

where *V*_*i*_ represents either an input or an output variable.

The studies of^56,57^ regarding the evaluation of different learning algorithms recommend the superior performance of the Levenberg–Marquardt algorithm, and hence, this algorithm is utilized in this research. In the architectural structure of the model, the structure of the hidden layer (i.e., the number of hidden layer/s and its neurons) affects the convergence of model learning. Based on the global approximation theorem^58^, a single hidden layer is considered for the models. Therefore, the initial structure of the model architecture is *6*_*Input*_-*nodes*_*Hidden*_-*1*_*Output*_. The number of *nodes*_*Hidden*_ depends on the number of inputs and output^59^. To achieve the final structure of the model architecture (i.e., determine the *nodes*_*Hidden*_), a detailed analysis will be performed in subsection “Determination of the final structure of models”.

### Performance evaluation metrics

Several error metrics have been used to evaluate the accuracy and compare the performance of the models developed in the current study. Table 2 lists the error metrics used, their mathematical expressions, and their ranges.

**Table 2:** The evaluation metrics for models' performance.

Indicator	Equation	Range, ideal
Mean absolute error	${\text{MAE}} = \frac{1}{{\text{T}}}\mathop \sum \limits_{{{\text{i}} = 1}}^{{\text{T}}} \left| {CD_{i, obs} - CD_{i, pre} } \right|$	(0, $+ \infty$), 0
Root mean squared error	${\text{RMSE}} = \sqrt {\frac{1}{{\text{T}}}\mathop \sum \limits_{{{\text{i}} = 1}}^{{\text{T}}} \left( {CD_{i, obs} - CD_{i, pre} } \right)^{2} }$	(0, $+ \infty$), 0
Relative root mean squared error	${\text{RRMSE}} = \frac{{{\text{RMSE}}}}{{\overline{{CD_{obs} }} }}$	Excellent: [0, 0.1] Good: (0.1, 0.2] Fair: (0.2, 0.3] Poor: (0.3, Inf)^60^
Coefficient of determination	${\text{R}}^{2} = \left( {\frac{{\mathop \sum \nolimits_{{{\text{i}} = 1}}^{{\text{T}}} \left( {CD_{i, obs} - \overline{{CD_{obs} }} } \right)\left( {CD_{i, pre} - \overline{{CD_{pre} }} } \right)}}{{\sqrt {\left[ {\mathop \sum \nolimits_{{{\text{i}} = 1}}^{{\text{T}}} \left( {CD_{i, obs} - \overline{{CD_{obs} }} } \right)^{2} } \right]\left[ {\mathop \sum \nolimits_{{{\text{i}} = 1}}^{{\text{T}}} \left( {CD_{i, pre} - \overline{{CD_{pre} }} } \right)^{2} } \right]} }}} \right)^{2}$	(0, 1), 1
Nash–Sutcliffe efficiency	${\text{NSE}} = 1 - \frac{{\mathop \sum \nolimits_{{{\text{i}} = 1}}^{{\text{T}}} \left( {CD_{i, obs} - CD_{i, pre} } \right)^{2} }}{{\mathop \sum \nolimits_{{{\text{i}} = 1}}^{{\text{T}}} \left( {CD_{i, obs} - \overline{{CD_{obs} }} } \right)^{2} }}$	($- \infty$, 1), 1
Performance index	${\text{PI}} = \frac{{{\text{RRMSE}}}}{{1 + {\text{R}}}}$	(0, 1), 0
A10-index	${\text{A}}10 = \frac{{{\text{M}}10}}{{\text{T}}}$	(0, 1), 1

*T* total of data.

$CD_{i, obs}$ and $CD_{i, pre}$ = The observed and predicted carbonation depth of the *i*th data, respectively.

$\overline{{CD_{obs} }}$ = The average of the observed carbonation depths.

M10 = The number of data in which their $CD_{obs}$/$CD_{pre}$ ratio fits in the range of 0.90–1.10.

### Cross-validation

**Figure 9:**
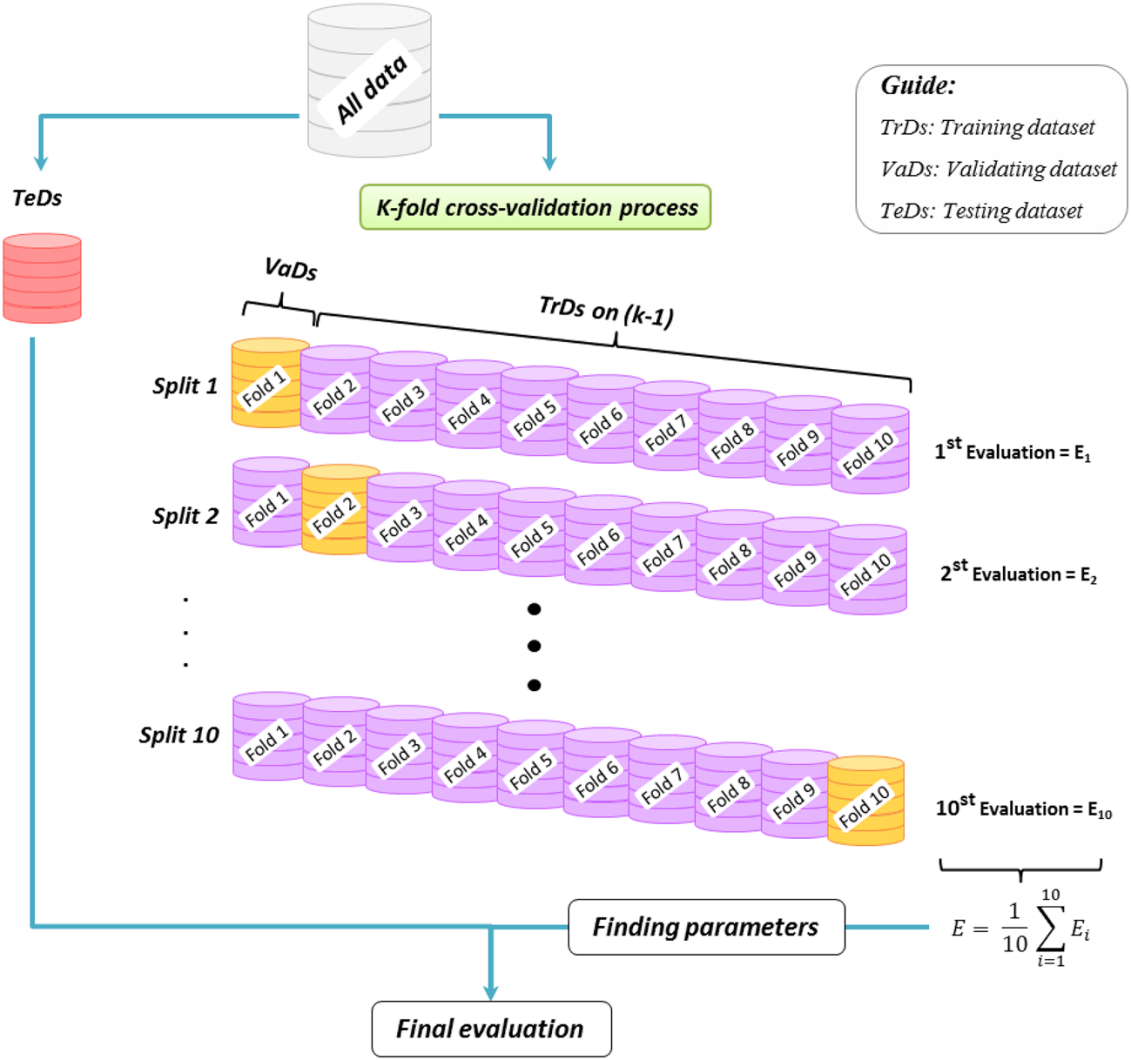
Schematic for ten-fold cross-validation analysis (this figure was created by the author using Microsoft PowerPoint 2016).

In the current study, a *K*-fold cross-validation analysis is developed to check the proposed models' ability to estimate new data and further reveal their skill assessment. Indeed, this analysis diminishes the overfitting issues associated with limited datasets and bias in the *TrDs* by its multiple evaluations. To this end, a *TeDs* is first taken from the cross-validation procedure to test the best-performing model. The dataset is then split into *K*-folds, and the model is trained on *K*-1 subsets and validated on the remaining subset. This process is continued until each of the *K*-folds has been used as the *VaDs*, and the average evaluation of the models is finally calculated^61^. Figure 9 depicts a schematic of generating datasets for the tenfold cross-validation analysis. This approach enhances the credibility of the proposed models and reduces the error rate in modeling by systematically validating its performance on distinct subsets of the data.

## Results and discussion

### Determination of the final structure of models

**Figure 10:**
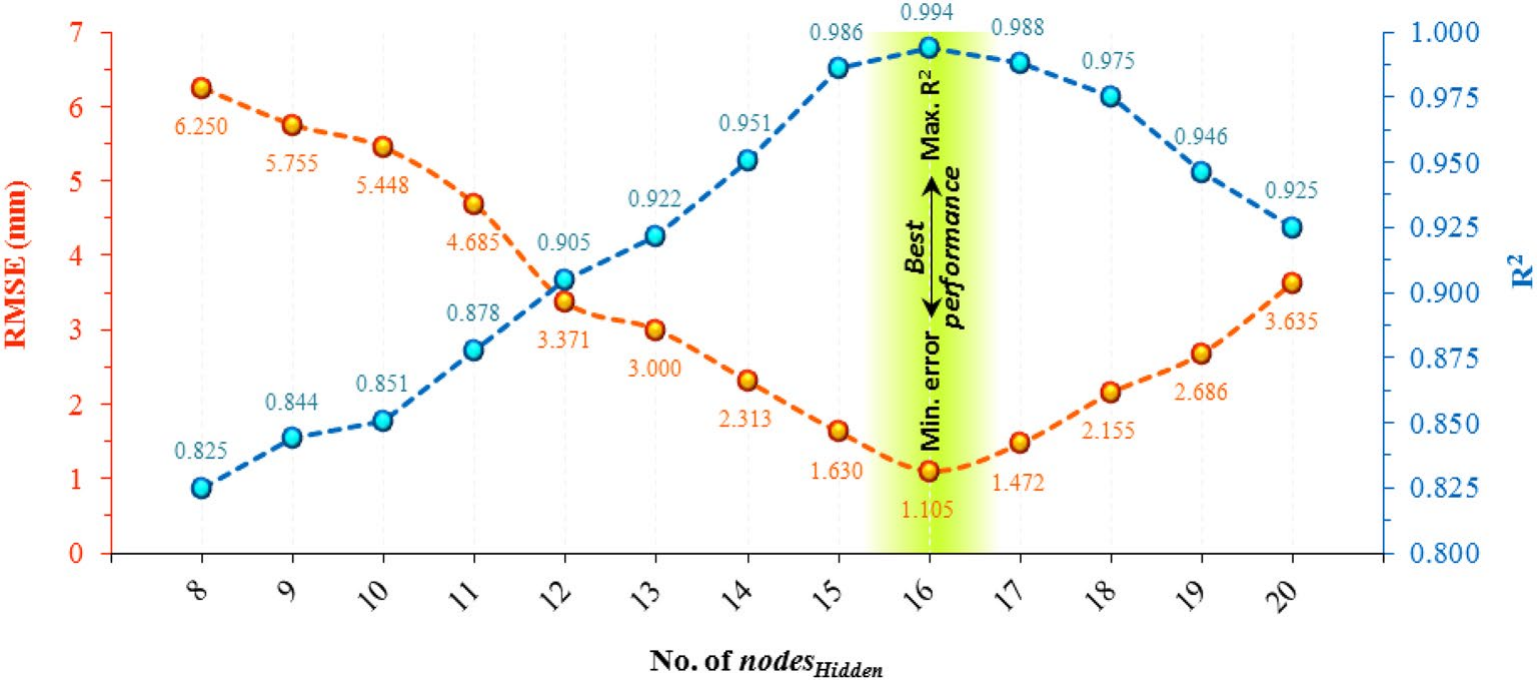
Variations of RMSE (red dotted line) and R^2^ (blue dotted line) against different No. of *nodes*_*Hidden*_. The performance with the lowest RMSE value and the highest R^2^ value is considered the best.

**Figure 11:**
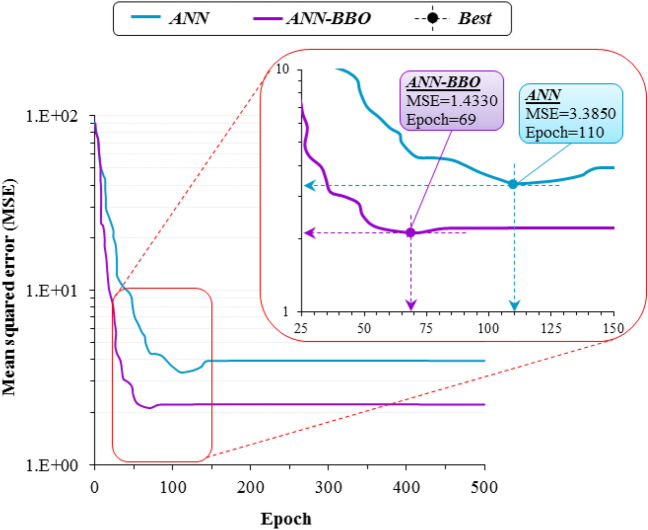
The convergence plot of the performance of ANN (blue line) and ANN-BBO (purple line) models.

To finalize the architectural structure of the 6-*nodes*_*Hidden*_-1 model (that is, determine *nodes*_*Hidden*_), an analysis is established during which the performance of models with different numbers of *nodes*_*Hidden*_ is evaluated. To have a fair comparison of the performance of the models, they are trained using an identical training dataset. Figure 10 depicts the training performance of different architectures by comparing the RMSE and R^2^ values. The best performance belongs to values closest to 0 and 1 for RMSE and R^2^, respectively. As depicted in the figure, the architecture with 16 nodes in the hidden layer outperforms other ones with the lowest RMSE (1.105 mm) and the highest R^2^ value (0.994). Accordingly, the *6*-*16*-*1* layout is considered the final architectural structure. Figure 11 illustrates the convergence performance of the models used in this study. As highlighted in the figure, the best performance for the hybrid model of ANN-BBO is achieved with MSE = 1.4330 at 69 epochs, while this value for the ANN model was MSE = 3.3850 at 110 epochs. This indicates the ability of the hybrid model of ANN-BBO compared to the single ANN model to achieve a lower MSE in fewer epochs, which means faster convergence plus improved performance.

Table 3 summarizes the settings of selected parameters to aid in presenting the information more meaningfully.

**Table 3:** Settings of selected parameters of ANN and BBO.

Method	Parameter		Setting
BBO	Population size		100
	Max emigration rate		1
	Habitat modification		1
	Mutation probability		0.005
ANN	Dataset partitioning (%)	Training	70
		Validating	15
		Testing	15
	No. of input parameter		6
	No. of hidden layer/nodes		1/16
	Learning algorithm		Levenberg–Marquardt
	Transfer function		Hyperbolic tangent
	Max. no. of epochs		500

### Performance of the developed models

**Figure 12:**
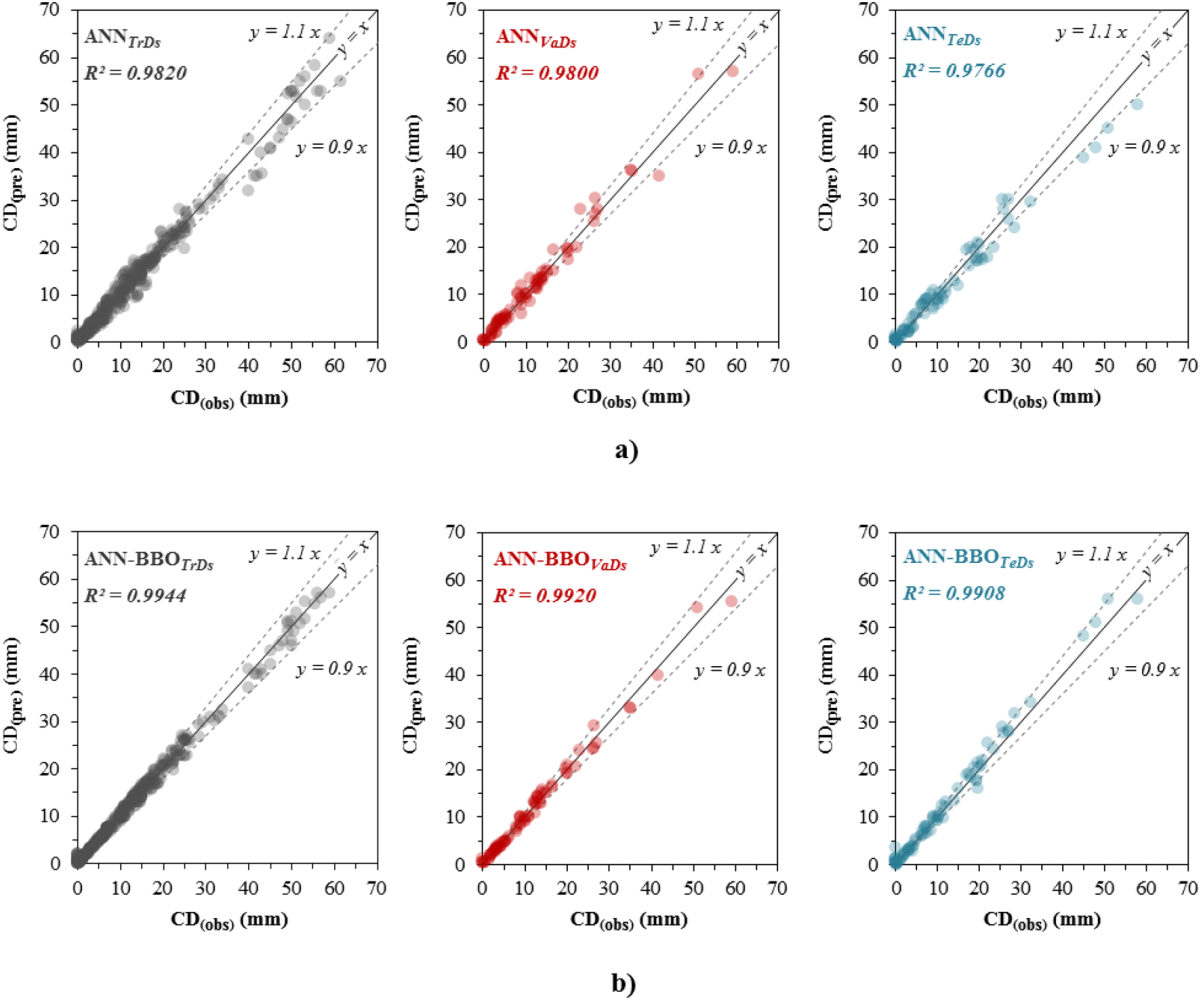
The observed v.s predicted values for the models of (**a**) ANN and (**b**) ANN-BBO (black, red, and blue colors refer to training, validating, and testing datasets, respectively). The dotted lines indicate ± 10% range compared to the best-fitting regression line (*y* = *x*).

The correlation between the predicted CD_(pre)_ values versus the observed CD_(obs)_ values on the *TrDs*, *VaDs*, and *TeDs* for the two developed models of ANN and ANN-BBO is depicted in Fig. 12. The R^2^ values by the ANN model in three datasets-*TrDs*, *VaDs*, and *TeDs* are obtained 0.9820, 0.9800, and 0.9766 while these values by ANN-BBO model are 0.9944, 0.9920, and 0.9908. The higher correlation between the observed and predicted values in the ANN-BBO model indicates that the data points are closer to the best-fitting regression line (*y* = *x*) than the single ANN model. Coherence between observation records and the most accurate predictions of CD of CCFA in three-*TrDs*, *VaDs*, and *TeDs* achieved by the proposed ANN-BBO model outperformed the ANN model. Furthermore, the minimum deviation from the ± 10% (black dotted lines) is obtained for the ANN-BBO model. It can be concluded that the developed ANN-BBO model has provided superior performance.

**Figure 13:**
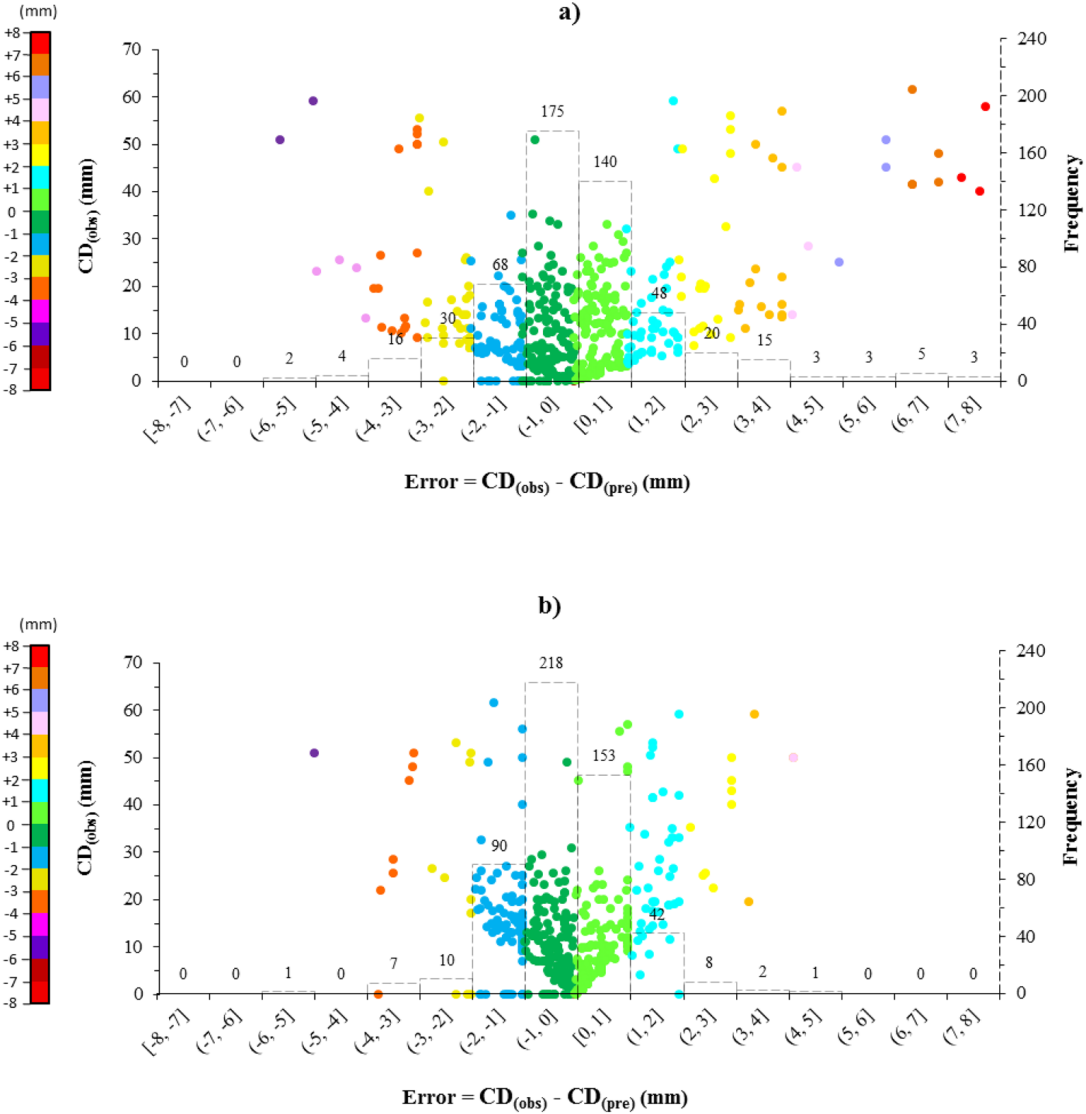
Plots of error distribution and histograms for the models of (**a**) ANN and (**b**) ANN-BBO. The frequency of errors around the zero value indicates better model performance.

A closer look at the error distribution of the models can help to better evaluate the performance of the models. Figure 13 illustrates the error distribution and error histograms of each developed model. By carefully examining the differences between CD_(obs)_ and CD_(pre)_ values, it is clearly visible that the ANN-BBO model estimated the depth of carbonate penetration into the CCFA with a narrower range of errors compared to the ANN model. For instance, regarding the frequency of the error intervals, 59% of the ANN predictions (315 data records out of 532) have errors within the range of (− 1 mm, 1 mm], while the corresponding percentage for the ANN-BBO predictions is 70% (371 data records out of 532), which indicates a decrease of 11% in the prediction errors by the proposed hybrid model. Indeed, it further elucidates the potential of the developed ANN-BBO model for more reliable predictive performance.

**Figure 14:**
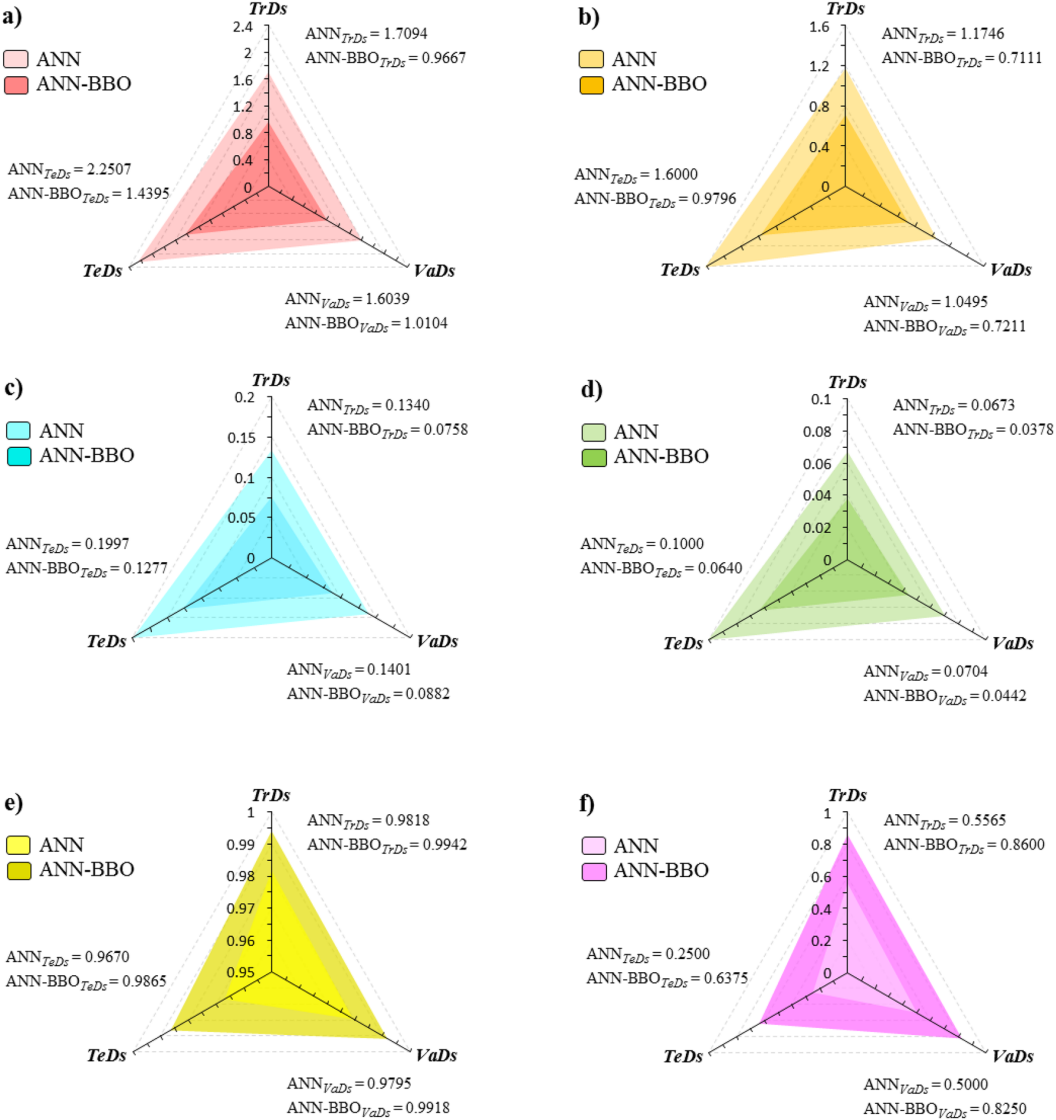
Comparative charts of evaluation metrics for ANN (light colors) and ANN-BBO (dark colors) models in three datasets. (**a**) RMSE, (**b**) MAE, (**c**) RRMSE, (**d**) PI, (**e**) NSE, and (**f**) A10-index.

Six evaluation metrics have been applied as radar charts to visualize the sensitivity analysis of the two developed models. Knowing the best performance limit of these metrics helps to evaluate them better, so it is worth mentioning that the best performance for RMSE, MAE, RRMSE, and PI (Fig. 14a–d) is obtained close to zero, while the best values for NSE and A10-index (Fig. 14e,f) are close to one. As can be seen from the figure, the ANN-BBO model shows less error for each of the four metrics of RMSE, MAE, RRMSE, and PI in all three datasets. For example, comparing the results of these four metrics indicates that the ANN-BBO model outperforms by a decrease of approximately 40%, 37%, and 36%, respectively, in the *TrDs*, *VaDs*, and *TeDs* compared to the single ANN model (compare Fig. 14a–d). In addition, the trend of improving the performance of the ANN-BBO model is also visible in the NSE metric (see Fig. 14e). In Fig. 14f, the A10-index appraises the data records that they meet the condition of 0.9 < CD_(obs)_/CD_(pre)_ < 1.1 so that a higher A10-index deduces the more reliability of the developed model. The results indicate that the ANN-BBO model experienced more A10-index in all three datasets than the ANN model, which means it is close to the best performance limit (i.e., closeness of the predicted values to the observed ones). Upon closer examination of the performance metrics across all datasets, it becomes apparent that the ANN-BBO model reveals significant improvement over the ANN model in error reduction. In summary, the results of all evaluation metrics are listed in Table 4. The results of evaluation metrics align with earlier results regarding the effectiveness of the ANN-BBO model in estimating the CD of CCFA.

**Table 4:** Comparison of evaluation metrics.

Model	Phase	Indicators						
		R^2^	RMSE (mm)	MAE (mm)	PI	NSE	A10-index	RRMSE
ANN	TrDs	0.9820	1.7094	1.1746	0.0673	0.9818	0.5565	0.1341_Good_*
	VaDs	0.9800	1.6039	1.0495	0.0704	0.9795	0.5000	0.1401_Good_
	TeDs	0.9766	2.2508	1.6184	0.1004	0.9669	0.2500	0.1997_Good_
	All	0.9795	1.7841	1.2219	0.0726	0.9716	0.5019	0.1446_Good_
ANN-BBO	TrDs	0.9944	0.9668	0.7112	0.0380	0.9942	0.8600	0.0758_Excellent_
	VaDs	0.9920	1.0105	0.7211	0.0442	0.9919	0.8250	0.0882_Excellent_
	TeDs	0.9908	1.4395	0.9796	0.0640	0.9865	0.6375	0.1277_Good_
	All	0.9929	1.0577	0.7530	0.0429	0.9900	0.8947	0.0858_Excellent_

*It represents the accuracy of the prediction according to the ranges in Table 2.

**Figure 15:**
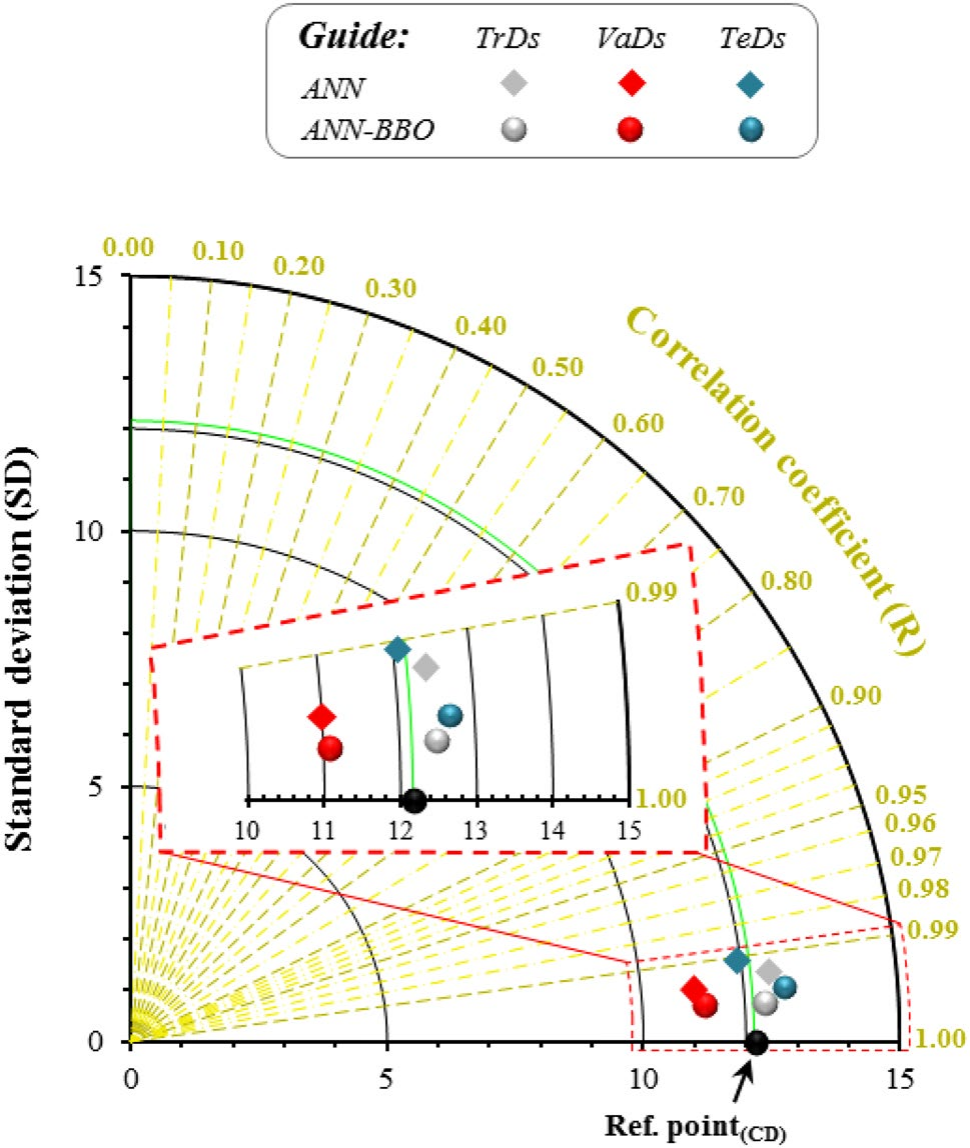
Taylor diagram of the developed models of ANN (rhombus) and ANN-BBO (circle) in three datasets. The closeness to the Ref. point indicates superior modeling performance.

Furthermore, two evaluation metrics- standard deviation (SD) and correlation coefficient (R) in polar coordinates have been integrated in the form of a Taylor diagram to clarify the correspondence between predicted and observed values. In this type of diagram, the closeness to the reference point (Ref. point) indicates superior modeling performance^62^. Figure 15 represents a Taylor diagram of the two proposed models of ANN and ANN-BBO on all three datasets-*TrDs*, *VaDs*, and *TeDs*. The performance of the models is visualized with a Ref. point. The results plotted in Fig. 15 express the strong correlation of two developed models with Ref. point, while the ANN-BBO model is nearer to the Ref. point in all three datasets. This means that the ANN-BBO model performs well compared to the ANN model. The results of the Taylor diagram prove that the hybrid proposed model of ANN-BBO can be more robust and efficient than the single ANN model in estimating the CD of CCFA.

### Cross-validation analysis

**Figure 16:**
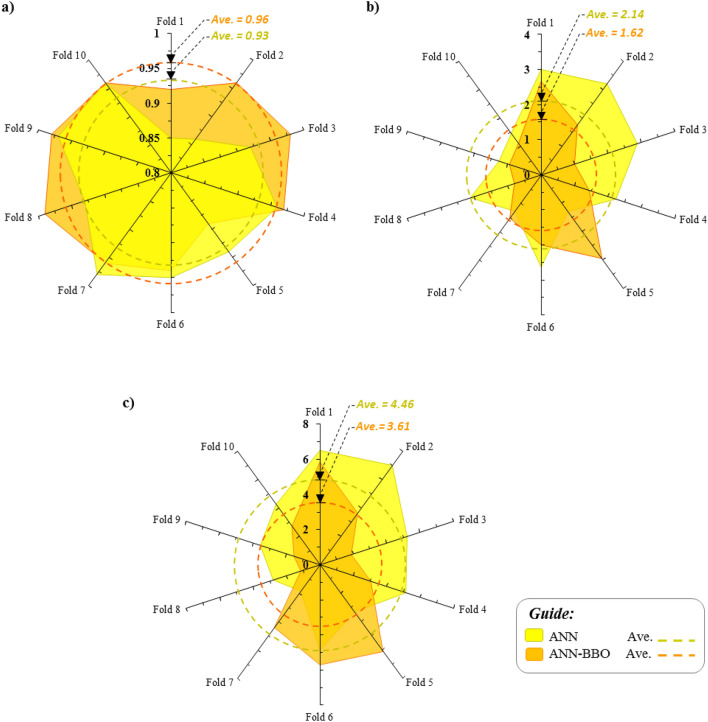
Ten-fold cross-validation analysis of the developed ANN and ANN-BBO models. (**a**) R^2^, (**b**) MAE, and (**c**) RMSE. The dotted lines indicate the average performance for the 10-folds considered.

To have a closer look at the reliability of the developed models' performance and their ability to generalize on new data, a *K*-fold cross-validation analysis has been performed. In this regard, an in-depth analysis has been repeated ten times during a process and compared using evaluation metrics of R^2^, MAE, and RMSE, as depicted in Fig. 16. The dotted lines denote the average value of evaluation metrics among all folds. By comparing the results obtained for the ten-fold analysis, it is observed that the average performance of R^2^ value for ANN and ANN-BBO models achieves 0.93 and 0.96, respectively. Moreover, the ANN-BBO model experiences an average MAE of 1.62 mm, whereas it has an error reduction of about 24% compared to the average MAE of the ANN model (2.14 mm). Similarly, a decreasing trend of 19% is observed for the average RMSE by the hybrid ANN-BBO model compared to the ANN model (see Fig. 16c). These findings infer that the developed ANN-BBO model has superior performance capability in generalizing to new datasets. In summary, Table 5 lists the results of the ten-fold cross-validation analysis.

**Table 5:** A list of the results of cross-validation analysis.

K-fold	ANN			ANN-BBO		
	R^2^	MAE (mm)	RMSE (mm)	R^2^	MAE (mm)	RMSE (mm)
1	0.85	3.00	6.52	0.92	2.66	5.86
2	0.86	3.20	7.00	0.96	1.76	3.65
3	0.92	2.88	5.22	0.98	1.00	1.88
4	0.96	2.24	5.15	0.97	1.44	3.05
5	0.94	1.30	3.33	0.89	2.95	6.12
6	0.95	2.63	4.86	0.94	2.00	5.70
7	0.98	1.28	1.85	0.96	1.52	4.42
8	0.93	2.12	2.80	0.99	0.80	1.14
9	0.97	1.26	3.57	0.98	0.95	1.55
10	0.96	1.45	4.25	0.96	1.10	2.72
Ave	0.93	2.14	4.46	0.96	1.62	3.61
Min	0.85	1.26	6.23	0.89	0.80	1.14
Max	0.98	3.20	7.00	0.99	2.95	6.12

### Influence of input variables

**Figure 17:**
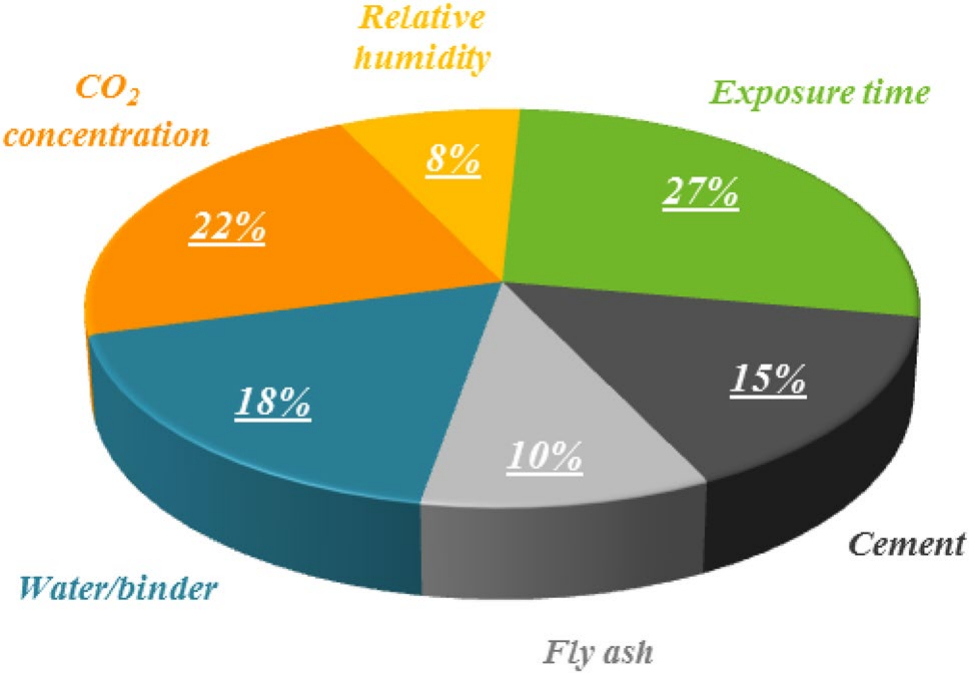
The influence of each input variable in the best-developed model (ANN-BBO) for estimating the CD of CCFA. The contribution rate of input variables is calculated by measuring the reduction in model performance when each input variable is excluded and not utilized in the modeling.

A comparative analysis is established to identify the effectiveness of each input variable in the best-developed model's performance in estimating the CD of CCFA. To this end, the contribution rate of input variables is calculated by measuring the degradation in model performance when each input variable is excluded and not utilized in the modeling (based on Alyuda NeuroIntelligence, Alyuda Research, Inc., Los Altos, California, USA). This approach identifies variables whose exclusion results in a meaningful decrease in model performance, which infers their influential role in predicting. Figure 17 illustrates the significance of the variables considered in the best-developed model (ANN-BBO). There is no doubt that all input variables contribute to the model performance, but their importance levels vary. As depicted in Fig. 17, the exposure time is the most influential variable affecting the CD of CCFA, with a contribution of 27%, which is consistent with the findings of previous studies^37,38^. Following that, CO_2_ concentration (22%), water/binder (18%), and cement (15%), respectively, are the next three important variables influencing the prediction of the CD in CCFA. Collectively, these four input variables (exposure time, CO_2_ concentration, water/binder, and cement) account for approximately 80% of the ability to estimate the CD of CCFA. The remaining input variables, including fly ash and relative humidity, are comparatively less influential, with contributions of 10% and 8%, respectively, toward estimating the CD of CCFA. By scrutinizing the results, it can be concluded that the two main variables in the CD of CCFA are related to the environmental condition, i.e., exposure time and CO_2_ concentration. Then, it is the turn of two mixture variables of water/binder and cement content. This finding can help engineers and researchers design and maintain RCSs against the carbonation phenomenon, a constant battle due to CO_2_ in the Earth's atmosphere.

### Comparison of the developed model with literature models

Without a doubt, the final and most essential step in AI studies is verifying the developed model through comparison with other AI techniques to specify the superiority and uniqueness of the chosen model. In this regard, it should not be overlooked that a fair comparison of models requires the studied subject and model output to be the same. Hence, a comparative analysis has been established to evaluate the best-developed model in the current study with similar recent studies in estimating the CD of CCFA with different AI techniques^35–38^. The accuracy of the models was evaluated by collecting their performance results in Table 6 using three evaluation metrics—R, MAE, and RMSE. Comparing the results indicates that the developed model outperformed the best literature models by providing a higher correlation coefficient (R) and lower error (MAE and RMSE). Consequently, the performed comparison confirms the improved accuracy and reliability of the developed hybrid ANN-BBO model in estimating the CD of CCFA.

**Table 6:** Comparing the best-developed model in the current study and previous studies.

Authors (year) [Ref.]	The best model	Evaluation metrics								
		R			MAE (mm)			RMSE (mm)		
		TrDs	TeDs	VaDs	TrDs	TeDs	VaDs	TrDs	TeDs	VaDs
Current study	ANN-BBO	0.9972	0.9954	0.9960	0.7112	0.9796	0.7211	0.9668	1.4395	1.0105
Huo et al. (2023)^38^	HEM-IV	0.996	0.975	–	0.687	1.788	–	1.204	2.978	–
Tran et al. (2023)^37^	XGB	0.9935	0.9884	–	0.5906	1.5218	–	1.4941	2.2725	–
Felix et al. (2021)^35^	ANN	0.9708	–	0.9572	–	–	–	2.6538	–	2.3348
Kellouche et al. (2019)^36^	ANN	0.9872	0.9468	0.9800	–	–	–	–	–	–

*HEM* hybrid ensemble machine learning, *IV* inverse variance, *XGB* extreme gradient boosting.

## Conclusions and future steps

One of the major challenges in the civil engineering sector is the durability of reinforced concrete structures against concrete carbonation and, thereby, corrosion risk. A countermeasure is the use of alternative cementitious materials in the form of sustainable concrete, which can be an effective solution to reduce the CD. Since the CD test is time-intensive and requires re-testing for changes in the ratio of materials and conditions, this study strives to provide an accurate and reliable AI approach for estimating the CD of CCFA. Hence, a hybrid model based on the metaheuristic optimization algorithm of BBO hybridized with the ANN model was developed to achieve a more efficient outcome than the single ANN model. The performance of the developed models was analyzed through evaluation metrics and ten-fold cross-validation, and finally, the best-developed model was compared with literature models. A concise summary of the findings is listed herein:

1. Evaluating all evaluation metrics indicated that the ANN-BBO model outperformed the single ANN model, with R^2^ values of 0.9944, 0.9920, and 0.9908 for *TrDs*, *VaDs*, and *TeDs*, respectively.
2. The error distribution and its histograms revealed that 59% of the ANN predictions experienced errors within the range of (− 1 mm, 1 mm], while the corresponding percentage for the ANN-BBO predictions was 70%, indicating a decrease of 11% in prediction errors by the proposed hybrid model. This indicates the potential of the developed ANN-BBO model in providing a more accurate model with a concentration of a narrower error range than the single ANN model.
3. Analyzing the cross-validation elucidated the reliability and generalizability of the ANN-BBO model using statistical indicators of R^2^, MAE, and RMSE.
4. Investigating the impact of input variables revealed that the most influential variable affecting the CD of CCFA was the exposure time, with a contribution of 27%, followed by CO_2_ concentration (22%), water/binder (18%), and cement (15%).
5. Finally, to verify the ANN-BBO model, a comparison was established with the results of literature models. Findings indicated that the best-developed model (ANN-BBO) provides, by far, the best performance in estimating the CD of CCFA compared to existing models.

The forward steps in the direction of expanding this study could include: (i) considering additional relevant variables and evaluating their effect on CD under different test conditions; (ii) expanding a more comprehensive database to strengthen further the achievement of a more reliable model with higher generalizability; and (iii) developing new AI-based models to provide more accurate models.

### Supplementary Information

Supplementary Information.

## Data Availability

The database used in this study is available in the supplementary file.

## Abbreviations

CD: Carbonation depth
FA: Fly ash
CCFA: Concrete containing fly ash
RCS: Reinforced concrete structure
CO_2_: Carbon dioxide
CaCO_3_: Calcium carbonate
Ca(OH)_2_: Calcium hydroxide
RH: Relative humidity
AI: Artificial intelligence
ANN: Artificial neural network
BBO: Biogeography-based optimization
HSI: Habitat suitability index
TrDs: Training dataset
VaDs: Validation dataset
TeDs: Testing dataset
MAE: Mean absolute error
RMSE: Root mean squared error
RRMSE: Relative root mean squared error
R^2^: Coefficient of determination
NSE: Nash–Sutcliffe efficiency
PI: Performance index
A10: A10-index
R: Correlation coefficient
SD: Standard deviation
